# Sulfonic Functionalized Polydopamine Coatings with pH-Independent Surface Charge for Optimizing Capillary Electrophoretic Separations

**DOI:** 10.3390/molecules29071600

**Published:** 2024-04-03

**Authors:** Wenwen Long, Mingyue You, Jieli Li, Yan Wang, Dan Wang, Xueping Tao, Li Rao, Zhining Xia, Qifeng Fu

**Affiliations:** 1 School of Pharmacy, Southwest Medical University, Luzhou 646000, China; 2 Chongqing Key Laboratory of High Active Traditional Chinese Drug Delivery System, Chongqing Medical and Pharmaceutical College, Chongqing 401331, China; 3 School of Pharmaceutical Sciences, Chongqing University, Chongqing 401331, China

**Keywords:** capillary electrophoresis, pH-independent electroosmotic flow, sulfonated polydopamine, capillary modification

## Abstract

Enhancing the pH-independence and controlling the magnitude of electroosmotic flow (EOF) are critical for highly efficient and reproducible capillary electrophoresis (CE) separations. Herein, we present a novel capillary modification method utilizing sulfonated periodate-induced polydopamine (SPD) coating to achieve pH-independent and highly reproducible cathodic EOF in CE. The SPD-coated capillaries were obtained through post-sulfonation treatment of periodate-induced PDA (PDA-SP) coatings adhered on the capillary inner surface. The successful immobilization of the SPD coating and the substantial grafting of sulfonic acid groups were confirmed by a series of characterization techniques. The excellent capability of PDA-SP@capillary in masking silanol groups and maintaining a highly robust EOF mobility was verified. Additionally, the parameters of sulfonation affecting the EOF mobilities were thoroughly examined. The obtained optimum SPD-coated column offered the anticipated highly pH-independent and high-strength cathodic EOF, which is essential for enhancing the CE separation performance and improving analysis efficiency. Consequently, the developed SPD-coated capillaries enabled successful high-efficiency separation of aromatic acids and nucleosides and rapid cyclodextrin-based chiral analysis of racemic drugs. Moreover, the SPD-coated columns exhibited a long lifetime and demonstrated good intra-day, inter-day, and column-to-column repeatability.

## 1. Introduction

Electroosmotic flow (EOF), as an essential driving force in the capillary electrophoresis (CE) separation of a wide range of analytes, and profoundly impacts the efficiency, selectivity, and reproducibility of CE [1,2,3]. However, variations in the surface chemistry of fused silica can engender inadequate EOF reproducibility, thereby impeding the wider acceptance and implementation of CE. Moreover, the surface charge density of fused-silica capillaries is notably susceptible to fluctuations in the pH of the background electrolyte (BGE), leading to a pronounced pH-dependent cathodic EOF that also impairs the repeatability of CE operations. The inherent pH-dependent cathodic EOF of the fused-silica capillaries is also not optimal for numerous CE applications. Furthermore, the strong adsorption tendency of positively charged analytes on the silica inner surface can further contribute to peak broadening, reduced reproducibility, and efficiency loss [2,4,5,6]. Therefore, it is essential to reduce the pH-dependence of the EOF mobility and establish an appropriate equilibrium between the EOF and electromigration to optimize CE methods for efficient and reproducible separations within a relatively short period of time.

To control the velocity and direction of EOF in CE, different techniques involving capillary inner surface modification have been introduced, such as dynamic coating, physical adsorption, and covalent bonding methods [3,7,8,9,10,11]. The dynamic coating method has gained distinction for its simplicity and practicality. However, dynamic modification frequently involves the regeneration of charge modifiers as pseudo-stationary phases present in the mobile phase, which may exert adverse impacts on the CE separation and detection processes [2]. Physically adsorbed coatings, such as successive multiple ionic–polymer layer (SMIL) coatings, have been widely used to regulate the EOF. Nevertheless, these non-covalent interaction-based modification strategies typically exhibit lower stability and limited durability [4,12]. By contrast, covalent coatings generally exhibit more robust modification effects and longer lifetimes. However, these coating protocols generally required rigorous and laborious preparation processes or encounter challenges related to reproducibility [11,12]. Moreover, it is difficult to eliminate completely and durably the pH-dependency of the surface charge of the capillary wall through traditional surface modification methods. Consequently, the limited pH-independence in modulating EOF could not facilitate the maintenance of reproducibility and introduced complexity to the methodological optimization of CE analysis [13]. Therefore, the exploration and development of more convenient and effective surface modification approaches for achieving pH-independent EOF are crucially significant for CE.

To enhance pH-independence in controlling EOF within CE, a few capillary modification methods using sulfonate-functionalized charge modifiers have been introduced, whose charge densities are actually constant over the whole pH range of the commonly used BGE [13,14]. Sun and co-workers developed a covalent coating method using the copolymers of neutral acrylamide and strongly anionic sodium-2-acrylamido-2-methyl-1-propanesulfonate with different molar fractions to achieve largely pH-independent and regulable cathodic EOF [13]. Katayama et al. utilized the SMIL coating composed of polybrene and dextran sulfate (DS) to modify capillaries, leading to an improvement in the pH-independence of the resulting cathodic EOF [15]. Despite these advancements, previously reported sulfonated functionalized capillaries generally still suffer from many issues such as complex and time-consuming fabrication processes, inadequate pH independence of EOF, and poor reproducibility.

Benefiting from the strong substrate-independent surface adhesive properties, versatile reactivity, relatively simple fabrication processes similar to physically adsorbed coatings, and favorable stability as covalent coatings, mussel-inspired polydopamine (PDA) and its derivative materials are highly suitable for various surface modification applications [16,17,18], including the inner wall modification of fused-silica capillaries [19]. Several functional PDA coated columns have been developed for CE or capillary electrochromatography (CEC) separations in different application fields [20,21], including the separation of small organic molecules [22,23,24,25], protein separation [26,27], chiral separation [28,29,30,31], etc. However, most existing PDA-based capillary modification strategies in CE, involving alkaline pH-induced oxidative polymerization of dopamine, often exhibit a poor surface masking effect and require an extended time ranging from over 20 h to several days, thus limiting reproducibility and practicality. The unmodified PDA coating adhered on the capillary inner wall generally exhibits a reduced EOF across a wide pH range due to partial masking of silanol groups, while an inversion of the EOF direction from cathodic to anodic occurs within the pH 5.0 to 4.0 range [32]. This phenomenon can be attributed to the isoelectric point of PDA, which is approximately at pH 4.0 [33]. In addition, despite the potential of PDA in regulating the surface charge of the capillary inner walls and achieving diverse EOFs having been preliminarily validated [25,32,34], the more interesting possibility of utilizing PDA for achieving pH-independent EOF in CE has not been explored.

**Figure 1:**
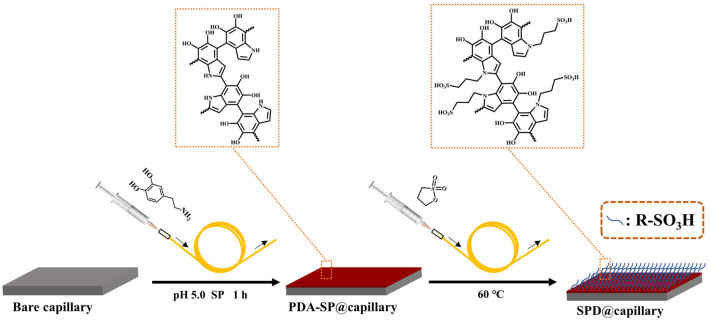
Scheme for preparation of PDA-SP@capillary and SPD@capillary.

Recently, new strategies involved in the fabrication of sulfonated PDA (SPD) coatings and periodate-induced rapid deposition of PDA coatings were proposed, which possess abundant surface grafted sulfonate groups and good surface masking effect on various substrates [35,36,37]. Herein, to reveal the potential use of SPD-based materials and periodate-induced PDA coating strategy to achieve highly pH-independent and stable cathodic EOF in CE, we reported a facile approach to modify rapidly SPD coatings inside a capillary with high surface coverage and uniformity via post-sulfonation of periodate-triggered PDA coating (Figure 1). A variety of measurements were comprehensively utilized to investigate the surface morphology, composition, and charge changes of various PDA coated capillaries, including Tris-induced PDA coated capillary (PDA-Tris@capillary), sodium periodate (SP)-triggered PDA coated capillary (PDA-SP@capillary), and sulfonated PDA-SP coated capillary (SPD@capillary). The successful immobilization of highly dense and uniform PDA-SP coating and the grafting of abundant sulfonic acid groups were confirmed. Subsequently, the excellent capability of PDA-SP@capillary in masking silanol groups and maintaining a highly stable EOF was demonstrated. The magnitude and pH-independence of the EOF mobility of SPD@capillary were further controlled by manipulating the sulfonation time and 1,3-propanesulfonate (1,3-PS) concentration. Under optimized sulfonation conditions, the SPD@capillary exhibited highly pH-independent and strong cathodic EOF behavior. Moreover, the extensive potential of the developed SPD@capillary in practical applications was further demonstrated through the efficient separation of aromatic acids and nucleosides and rapid CD-based chiral analysis of racemic drugs.

## 2. Results

### 2.1. Characterization of PDA and SPD Coatings

#### 2.1.1. FESEM

**Figure 2:**
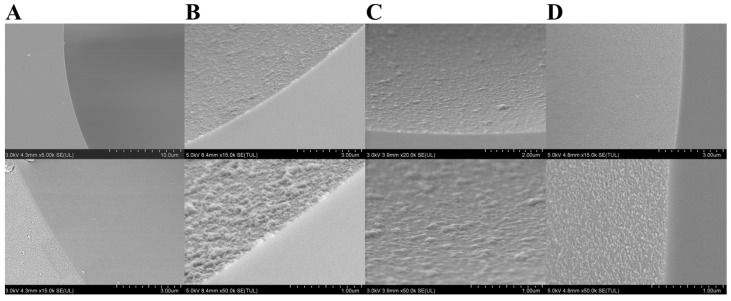
FESEM images of bare capillary (**A**), PDA-SP@capillary (**B**), SPD@capillary (**C**), and PDA-Tris@capillary (**D**).

The surface morphology of bare column, PDA-Tris@capillary, PDA-SP@capillary, and SPD@capillary were observed in detail using FESEM. As illustrated in Figure 2A, the bare column exhibited a smooth inner surface, while the dense layers with some granular aggregates fully covering the inner surface could be clearly observed on PDA-SP@capillary, fabricated after 1 h of SP-triggered dopamine polymerization reaction (Figure S1). Moreover, the microstructural morphology of the coatings from different cross-sections of a PDA-SP@capillary displayed a high level of agreement, indicating excellent uniformity of PDA-SP coatings (Figure S1). After long-term washing treatment with HCl (pH 2.0) and NaOH (pH 10.0) solutions for 12 h, no significant changes in the morphology and surface coverage of the PDA-SP coating on the capillary inner wall were observed (Figure S2A,B), indicating its excellent chemical stability and wide pH tolerance. The high surface coverage of PDA-SP coating ensures an effective masking of the silanol groups of the capillary (Figure 2B). After post-sulfonation treatment of PDA-SP@capillary, the surface morphology and coverage of the PDA coating had no significant change (Figure 2C), verifying the maintenance of the good surface modification effect in SPD@capillary. By contrast, the coverage of the PDA-Tris coating was relatively low, and could not cover the inner surface of the PDA-Tris@capillary (Figure 2D). Additionally, significant differences were observed in the microstructural morphology and surface coverage of PDA-Tris coatings among different cross-sections within a single capillary (Figure S3). This indicated their poor coverage which could be ascribed to the weak and unsustainable oxidation capability of dissolved oxygen. The above characterization results of FESEM fully demonstrate that the SP-induced deposition method can effectively improve the coverage, uniformity, and preparation efficiency of the PDA coating on the capillary inner wall, consistent with previous research findings [35,36].

#### 2.1.2. ATR-FT-IR and XPS

**Figure 3:**
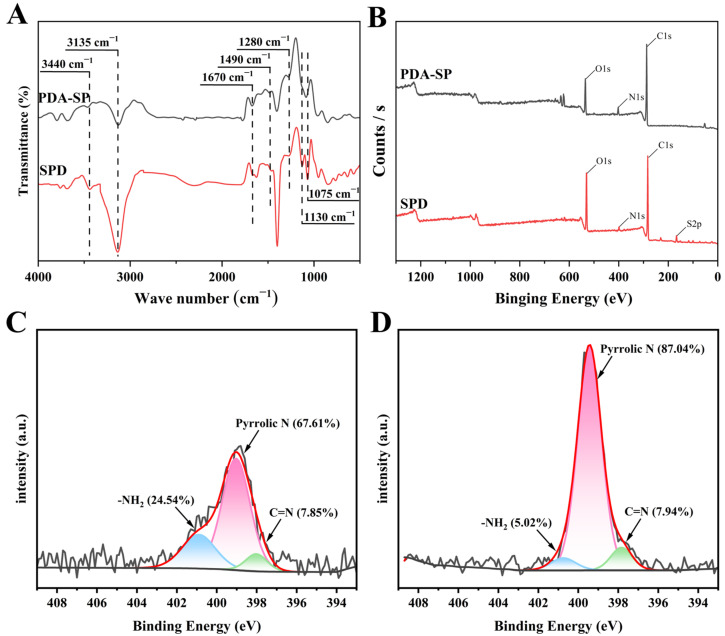
ATR-FT-IR (**A**), XPS survey spectra (**B**) of PDA-SP and SPD coatings and N1s peaks of PDA-SP (**C**) and SPD (**D**).

The surface functional groups of the PDA-SP and SPD coatings on the quartz plates were analyzed using ATR-FT-IR and XPS measurements (Figure 3). As shown in Figure 3A, the PDA-SP coating exhibited absorption peaks at approximately 3135 and 3440 cm^−1^, corresponding to the stretching vibrations of the catechol -OH and N-H groups of PDA [32,38]. Additional absorption peaks were observed at around 1670 cm^−1^, 1490 cm^−1^, and 1280 cm^−1^, corresponding to the C=O stretching, benzene skeleton vibration, C-O stretching and primary amine vibration from PDA [32,39]. Following the post-sulfonation treatment of the PDA-SP coating, the absorption peaks associated with PDA were well preserved and new absorption peaks emerged at approximately 1075 and 1130 cm^−1^, which can be attributed to the symmetric and asymmetric stretching vibrations of sulfonic groups [40]. On the other hand, the full XPS spectrum also showed that a new S2p sulfur atom signal at 167 eV appeared on the surface of the SPD coating (Figure 3B), further demonstrating the successful grafting of sulfonic groups on the SPD coating. Furthermore, the N1s spectra showed higher levels of non-dissociated pyrrolic nitrogen in SPD compared to PDA-SP, along with a notable decrease in the proportion of primary amine groups in SPD. Collectively, the characterization results from ATR-FT-IR and XPS confirm the successful attachment of sulfonic acid groups to the SPD coating, and suggest that sulfonation treatment results in a significant decrease in the number of positively charged amino groups, which contributes to enhancing the pH-independent surface charge properties of SPD coatings.

### 2.2. Influence of Number of PDA-Tris and PDA-SP Coatings on EOF Mobility

The important pre-requisites for achieving pH-independent EOF through the surface modification method is effectively suppressing the ionization of silanol groups on the capillary inner surface. Figure S4 illustrates the influence of the number of repeated PDA-Tris and PDA-SP coatings on their EOF mobilities under alkaline conditions, thereby ensuring thorough dissociation of potentially incompletely shielded silanol groups. With an increase in the number of PDA-Tris coatings from one to three, the magnitude of the cathodic EOF of PDA-Tris@capillary continuously decreases, indicating that the pH dependence of the underlying quartz substrate is consistently maintained and the ionization of the silanol groups is not completely inhibited. By contrast, a single PDA-SP coating achieves significant suppression of the EOF. After repeated coating treatments, the EOF mobility of PDA-SP@capillary remains almost the same as that of a single coating. These results suggest that due to its significantly improved coverage and uniformity revealed by FESEM, the PDA-SP coating exhibits a significantly superior silanol masking ability for modifying the capillary inner surface compared to the PDA-Tris coating, and can effectively mask the ionization of silanol groups in a single coating.

### 2.3. EOF Stability and Reproducibility of PDA-Tris@capillary and PDA-SP@capillary

Next, the EOF stability and reproducibility of PDA-Tris@capillary and PDA-SP@capillary were systematically investigated by observing the possible changes in EOF mobilities of the columns from the same batches during successive injections of the neutral marker DMSO under both acidic (pH 4.0) and alkaline (pH 8.0) conditions. As shown in Figure S5, the EOF of PDA-SP@capillary was cathodic at pH 8.0 and anodic at pH 4.0. This behavior can be attributed to the co-existence of amine, hydroxyl, and carboxyl groups on the surface of the PDA-SP coating [35]. Furthermore, the magnitude of the cathodic or anodic EOF remained relatively constant after 30 consecutive injections, and there were no significant differences observed in the EOF between PDA-SP@capillaries fabricated from different batches. In contrast, significant differences in EOF magnitude and relatively poor cathodic EOF stability at pH 8.0 were observed between different batches of PDA-Tris@capillaries. These results indicate that the EOF mobilities of PDA-SP@capillary are stable and reproducible, which are superior to those of PDA-Tris@capillary and coincide with the characterization results of FESEM.

### 2.4. EOF Mobilities of SPD@capillary

**Figure 4:**
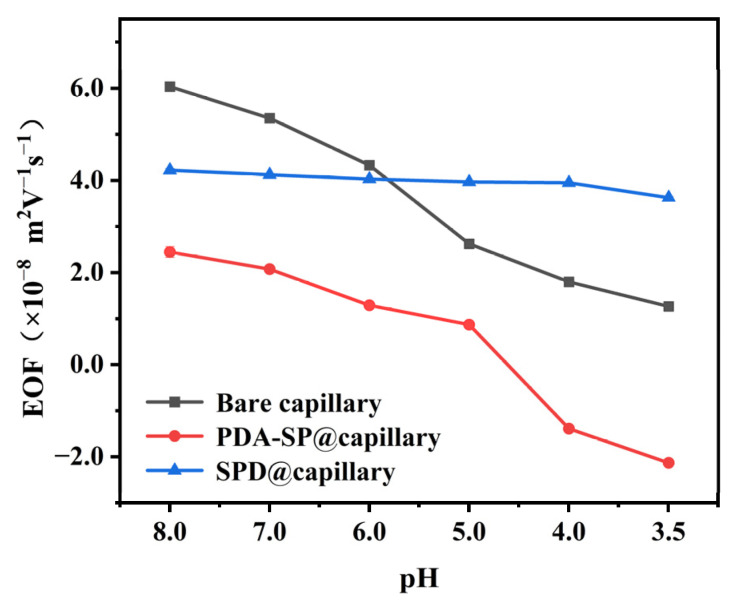
The EOF mobilities of bare capillary, PDA-SP@capillary, and optimum SPD@capillary at different pHs. Conditions: background electrolyte, 20 mM acetic buffer; EOF marker, DMSO; applied voltage, 20 kV; sample injection pressure and time at 35 mbar and 5 s; UV detection at 200 nm; temperature, 20 °C; capillary column with 33.0 cm (24.5 cm effective length), 75 μm i.d. and 375 μm o.d.

Despite its excellent silanol masking capability and good coating stability, the as-prepared PDA-SP@capillary exhibits undesirable pH-dependent EOF characteristics due to the presence of diverse charged functional groups on the surface of the PDA-SP coating. In order to enhance the pH independence of the EOF, we further subjected PDA-SP@capillary to post-sulfonation modification using 1,3-PS. This treatment resulted in the grafting of sulfonic acid groups with consistent charged properties and a notable reduction in unstable charged amino groups on the inner surface of SPD@capillary. Consequently, the EOF mobilities can be adjusted by varying parameters in the post-sulfonation reaction, such as sulfonation time and 1,3-PS concentration. As shown in Figure S6, strong cathodic EOF with optimal pH-independence can be achieved at 60 mM 1,3-PS concentration and 24 h sulfonation. Overall, Figure 4 offers a comprehensive depiction of the pH-dependent EOF properties of optimal SPD@capillary, PDA-SP@capillary, and unmodified columns, highlighting the significant impact of post-sulfonation treatment on achieving pH-independent EOF mobility.

### 2.5. Applications of SPD@capillary

The aforementioned experimental findings provide clear evidence that achieving a cathodic EOF with significantly improved pH independence and controllable magnitude is highly feasible by effectively controlling the post-sulfonation parameters of SPD coatings. In order to evaluate the practicality of SPD@capillaries in CE, further investigation was conducted to examine their separation performance for complex samples using two representative model compounds, i.e., aromatic acids and chiral drugs.

#### 2.5.1. High-Efficiency Separation of Aromatic Acids

**Figure 5:**
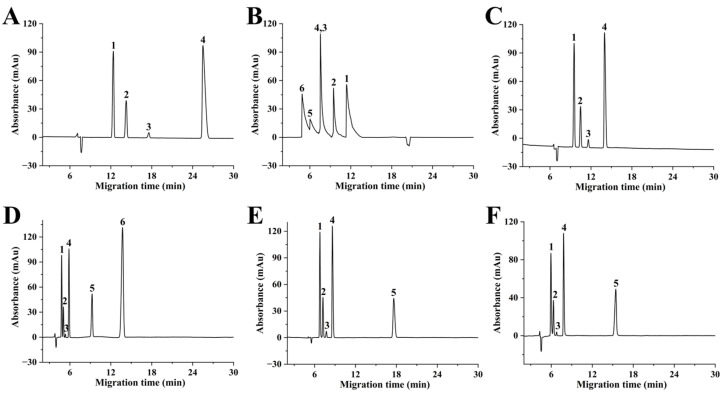
Electropherograms of six aromatic acids on bare capillary (**A**), PDA-SP@capillary (**B**). SPD@capillaries produced with sulfonation time of 24 h using 30 mM 1,3-PS (**C**), 60 mM 1,3-PS (**D**), 90 mM 1,3-PS (**E**), and SPD@capillary (**F**) produced with sulfonation time of 30 h using 60 mM 1,3-PS. (1) 4-hydroxybenzoic acid, (2) 2-phenylpropionic acid, (3) ascorbic acid, (4) benzoic acid, (5) 4-nitrobenzoic acid, and (6) salicylic acid. Conditions: background electrolyte, 20 mM acetic buffer, pH 4.0; capillary column with 48.5 cm (40.0 cm effective length), 75 μm i.d. and 375 μm o.d. Other experimental conditions are the same as Figure 4.

Figure S7 illustrates the effect of buffer pH on the separation efficiency of six aromatic acids on a bare column under optimal CE conditions, i.e., using a 20 mM acetic buffer at 20 kV. While four aromatic acids can achieve efficient baseline separation under an optimal buffer pH of 4.0, 4-nitrobenzoic acid (pKa = 3.42) and salicylic acid (pKa = 3.01) are not eluted, and no detectable peaks are observed within 30 min (Figure 5A). Indeed, even when the separation duration was extended up to 60 min, they remained undetected. This loss of peaks may be caused by the weak cathodic EOF generated by the unmodified column, which opposes the migration of the analytes with strong negative charges. By contrast, the PDA-SP@capillary, where analytes migrate in the same direction as the anodic EOF, shows rapid elution and reversal of elution order for all six aromatic acids (Figure 5B). However, these conditions also result in significant tailing and peak overlapping, potentially due to electrostatic interactions between the positively charged PDA coatings and the negatively charged analytes. Conversely, a notable enhancement in column efficiency and reduced analysis time were observed when employing SPD@capillaries with different levels of sulfonation for separating acidic compounds (Figure 5C–F). Remarkably, the optimized SPD@capillary enables the attainment of baseline separation for all six aromatic acids with high plate numbers (up to 8.7 × 10^4^ plates/m for benzoic acid) within just 15 min (Figure 5D). This improvement can be attributed to the strong cathodic EOF and effective suppression of adsorption effects on the negatively charged SPD coating.

#### 2.5.2. Rapid CDs-Based Chiral Analysis

**Figure 6:**
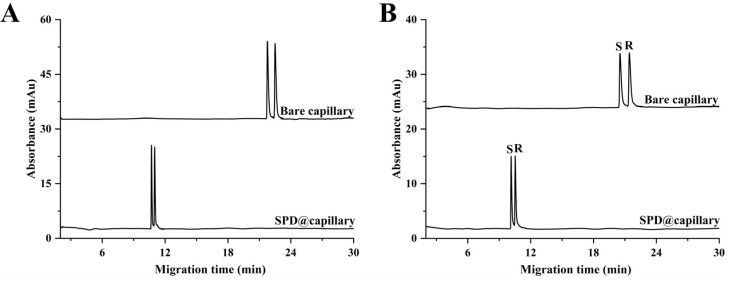
Electropherograms of (**A**) isoproterenol and (**B**) ofloxacin enantiomers on bare capillary and SPD@capillary at the same conditions. Background electrolyte, methanol–100 mM phosphate buffer (5/95, *v*/*v*), pH 4.0, 60 mM HP-β-CD; applied voltage, 25 kV; sample injection pressure and time at 50 mbar and 5 s; UV detection at 200 nm for isoproterenol and 290 nm for ofloxacin; temperature, 20 °C; capillary with 58.5 cm total length (50.0 cm effective length), 50 μm i.d. and 375 μm o.d.

Next, the potential application of SPD@capillary for CD-based rapid chiral analysis of racemic drugs is further explored. As shown in Figure 6, the baseline separation of the model racemic compounds, namely ofloxacin and isoproterenol enantiomers, could be achieved on the unmodified column using a relatively high concentration of HP-β-CD at an optimized buffer pH of 4.0 (Figure S8). However, their elution rates were slow, and the analysis time exceeded 20 min, which can be attributed to the limited driving force provided by the weak cathodic EOF of the bare fused-silica capillary under acidic conditions. By contrast, under optimized CE conditions (Figure S9), rapid baseline separation of the two model enantiomers with high separation efficiency was successfully achieved on the optimum SPD@capillary using the same concentration of HP-β-CD, as mentioned above (Figure 6). Notably, the migration times of the two model enantiomers were substantially reduced by more than two times, from approximately 21 min to 10 min. This significant improvement in chiral analysis efficiency can be ascribed to the strong and stable cathodic EOF provided by SPD@capillary. Specifically, the significantly enhanced EOF mobility imparted higher apparent mobility to the chiral analytes, thereby contributing to a substantial reduction in analysis time. Consequently, the aforementioned comparative analysis on the separation effects of aromatic acids and chiral drugs hugely demonstrated the immense potential of the developed SPD@capillary for achieving rapid and high-efficiency CE analysis.

#### 2.5.3. High-Efficiency Separation of Nucleosides

**Figure 7:**
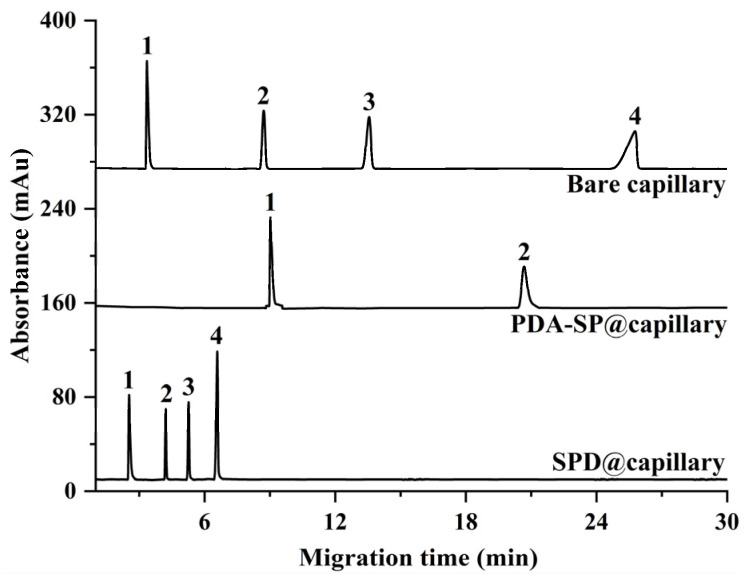
Electropherograms of four nucleosides on bare capillary, PDA-SP@capillary, and SPD@capillary. (1) cytosine, (2) uridine, (3) cytidine, (4) adenosine 5′-monophosphate. Conditions: All experimental conditions are the same as Figure 5.

To further substantiate the applicability of the SPD@capillary in the domain of biomolecule analysis, Figure 7 compares the separation performance of bare capillary, PDA-SP@capillary, and SPD@capillary for nucleosides under identical conditions. In the case of PDA-SP@capillary, within 30 min, a loss of peaks corresponding to cytidine and adenosine 5′-monophosphate was observed, which can be attributed to the weak anodic EOF produced by the PDA coating under acidic conditions (Figure 4), leading to migration of these analytes against the EOF direction. The bare capillary exhibits cathodic EOF consistent with the analyte’s movement. However, due to its weaker cathodic EOF strength, the nucleosides elute at a significantly slower rate, culminating in a requirement of 30 min to attain adequate baseline separation. In contrast, SPD@capillary maintains a substantial EOF magnitude under acidic conditions, which dramatically enhances the migration velocities of the analytes. Consequently, all four nucleosides exhibit baseline separation within 7 min, coupled with high separation efficiency (up to 9.4 × 10^4^ plates/m for cytidine), which highlights the potential applicability of SPD@capillary for rapid and efficient biomolecule separation.

### 2.6. Repeatability and Stability of SPD@capillary

The important prerequisites for the practical application of SPD@capillary are repeatability and stability. As indicated in Table 1, the relative standard deviations (RSDs) of migration time for separating the aromatic acids were all below 5% for both intra-day and inter-day analysis, as well as across different columns. Furthermore, after conducting CE separation of the aromatic acids 30 times, there were no notable differences observed in both selectivity and migration time (Figure S10). These findings collectively demonstrate that the fabricated SPD@capillary possesses good repeatability and excellent stability.

## 3. Materials and Methods

### 3.1. Reagents and Chemicals

Dopamine hydrochloride, tris (hydroxymethyl) aminomethane (Tris), sodium periodate (SP), 1,3-propanesulfonate (1,3-PS), and aqueous ammonia were obtained from Aladdin Reagent Co., Ltd. (Shanghai, China). Salicylic acid, benzoic acid, 2-phenylpropionic acid, 4-nitrobenzoic acid, ascorbic acid, and 4-hydroxybenzoic acid were all purchased from Adamas Reagent Co., Ltd. (Shanghai, China). Hydroxypropyl-beta-cyclodextrin (HP-β-CD) with molecular weight of 1540 was from Titan Scientific Co., Ltd. (Shanghai, China). Ofloxacin and isoproterenol were purchased from Sopo Biological Technology Co., Ltd. (Guangzhou, China). Ethanol, dimethyl sulfoxide (DMSO), disodium hydrogen phosphate (Na_2_HPO_4_), acetic acid, phosphoric acid, hydrochloric acid (HCl), and acetonitrile (ACN) were supplied by KeLong Chemical Reagent Co., Ltd. (Chengdu, China). Fused-silica capillaries (75 and 50 μm i.d. × 375 μm o.d.) were purchased from Ruifeng Chromatographic Devices Co., Ltd. (Yongnian, HanDan, China). The ultrapure water utilized in this study was purified using an ultrapure water system (AWL-1002-H, Aquapro, Chongqing, China).

### 3.2. Instrumentation

The Agilent 7100 3D CE system (Agilent Technologies, Waldbronn, Germany) with a diode array detector was utilized for CE experiments. The inner surface morphology of the bare column and as-prepared functionalized PDA coated columns were characterized using FESEM (SU-8010, Hitachi, Tokyo, Japan). The surface component analysis was performed by ATR-FT-IR spectroscopy (Nicolet IS10, Thermo Fisher Scientific, Waltham, MA, USA) and X-ray photoelectron spectroscopy (XPS) (ESCALAB 250Xi, Thermo Fisher Scientific, Waltham, MA, USA).

### 3.3. Preparation of PDA Coated Columns

The PDA-SP@capillary was fabricated by filling the column with a dopamine/SP mixed solution and allowing them to react for the desired time [35]. Typically, 40 mg SP and 20 mg dopamine were co-dissolved in 10 mL sodium acetate buffer (50 mM, pH 5.0) and thoroughly mixed using ultrasound treatment. The obtained solution was then injected into an acid- and base-activated fused silica capillary using a syringe pump. The column was then sealed at both ends and allowed to react for 1 h at room temperature. The resulting capillary was rinsed with ultrapure water for 10 min and dried with nitrogen gas to obtain the PDA-SP@capillary. In addition, the traditional alkaline pH-induced method was used to prepare the PDA-Tris@capillary as a control. In brief, 20 mg of dopamine was dissolved in 10 mL of Tris-HCl buffer solution (50 mM, pH 8.5) and injected into a pre-activated capillary using a syringe pump at 0.05 mL/h for 20 h at room temperature. The column was then rinsed with ultrapure water for 10 min and dried with nitrogen gas to obtain the PDA-Tris@capillary.

The SPD@capillary was prepared by post-sulfonation of PDA-SP@capillary using a certain concentration of 1,3-PS [41,42]. The amine group-rich PDA-SP coating could be covalently functionalized with sulfonic groups based on the ring-opening reaction between amine groups and 1,3-PS under mild conditions, whose preparation procedures are schematically shown in Figure 1. Typically, 60 mM 1,3-PS and 35 mM aqueous ammonia were added dropwise to anhydrous ethanol. After thoroughly mixing, the solution was injected into a nitrogen-dried PDA-SP@capillary using a syringe pump. The column was then sealed at both ends and allowed to react at 60 °C for 24 h. Subsequently, the sulfonated column was rinsed with absolute ethanol for 10 min and dried with nitrogen gas to obtain the SPD@capillary.

### 3.4. Sample and Buffer Solutions Preparation

Standard stock solutions were prepared for each analyte by individually dissolving them in ultrapure water or anhydrous ethanol, at a concentration of 5.0 mg/mL. The running buffers used for measuring EOF and carrying out CE separation of aromatic acids were prepared by dissolving the appropriate quantities of sodium acetate in ultrapure water. The pH of the running buffers was controlled using acetic acid or a 0.1 M NaOH solution. The running buffers for chiral CE analysis were prepared by dissolving HP-β-CD in phosphate buffer solutions composed of Na_2_HPO_4_, in the desired concentrations. All the sample and buffer solutions were filtered with 0.45 μm membrane filters and degassed with ultrasonication prior to use.

### 3.5. CE Procedures

The CE separation procedures were performed using the as-prepared columns. Prior to commencing electrophoresis each day, the columns were flushed with ultrapure water for 5 min, followed by running buffer for 10 min. Subsequently, after the completion of daily electrophoresis, the capillaries were filled with ultrapure water and stored at room temperature. The temperature during sample separation was maintained at 20 °C. Injection of all samples took place at a pressure of 35/50 mbar for a period of 5 s, with detection occurring at a wavelength of 200 nm and 290 nm.

## 4. Conclusions

This study presents a novel approach for effectively achieving the pH-independence of EOF using a sulfonated PDA coating. The developed PDA-SP coating, which firmly attaches to the inner wall of the capillary with exceptional coverage and uniformity, combines the advantages of physical adsorption and covalent bonding techniques to effectively mask the silanol groups on the capillary inner wall. Through a post-sulfonation treatment of the PDA-SP@capillary, the cathodic EOF with significantly enhanced pH independence and controllable magnitude, can be readily achieved by simply modifying the sulfonation parameters. The optimized SPD@capillary demonstrates highly pH-independent cathodic EOF and exhibits promising potential in enhancing the separation efficiency of acidic complex samples and biomolecules as well as enabling rapid chiral analysis. This cost-effective and facile PDA-based capillary coating approach holds significant promise for enhancing the reproducibility and efficiency of CE analysis.

## Figures and Tables

**Table 1:** Repeatability of the optimum SPDA@capillary.

Analytes	Migration Time (RSD%)		
	Intra-Day (*n* = 5)	Inter-Day (*n* = 5)	Column-to-Column (*n* = 3)
4-hydroxybenzoic acide	1.74	1.35	1.22
2-phenylpropionic acid	1.89	1.58	1.37
ascorbic acid	1.97	0.67	1.47
benzoic acid	2.59	1.95	1.84
4-nitrobenzoic acid	2.49	2.06	2.48
salicylic acid	3.54	2.39	3.46

## Data Availability

Data are contained within the article or Supplementary Materials.

## Supplementary Materials

The following supporting information can be downloaded at: https://www.mdpi.com/article/10.3390/molecules29071600/s1, Figure S1: FESEM images of the cross-sections of PDA-SP@capillary from the same batches, Figure S2: FESEM images of the inner wall of PDA-SP@capillary after washing treatment with HCl (pH 2.0) and NaOH (pH 10.0) solutions for 12 h, Figure S3: FESEM images of the inner wall of PDA-Tris@capillary from the same batches, Figure S4: Effect of the number of repeated coatings of PDA-SP@capillary and PDA-Tris@capillary on their EOF mobilities at pH 8.0, Figure S5: EOF stability of PDA-SP@capillary at pH 8.0 and pH 4.0 and PDA-Tris@capillary at pH 8.0 and pH 4.0, Figure S6: Effect of 1,3-PS concentration and sulfonation time on EOF mobilities of SPD@capillary, Figure S7: Electropherograms of six aromatic acids on bare column at different pHs, Figure S8: Electropherograms of isoproterenol and ofloxacin on bare column at different pHs, Figure S9: Electropherograms of isoproterenol on SPD@capillary at different buffer concentration, methanol concentration and applied voltage, Figure S10: Electropherograms of six aromatic acids of different runs on SPD@capillary.
