# Supplementary material for: Sulfonic Functionalized Polydopamine Coatings with pH-Independent Surface Charge for Optimizing Capillary Electrophoretic Separations
Source: Molecules. 2024 Apr 3;29(7):1600. doi: 10.3390/molecules29071600 (PMC11013714; doi:10.3390/molecules29071600)
Supplement: Supplementary file 1 [file molecules-29-01600-s001.zip › molecules-2940501-supplementary.pdf]

Supplementary data

Sulfonic Functionalized Polydopamine Coatings with pH-Independent Surface Charge for Optimizing Capillary Electrophoretic Separations

Wenwen Long [1,†], Mingyue You [1,†], Jieli Li [1], Yan Wang [1], Dan Wang [1], Xueping Tao [1], Li Rao [2,*], Zhining Xia [3,*] and Qifeng Fu [1,*]

[1] School of Pharmacy, Southwest Medical University, Luzhou 646000, China
[2] Chongqing Key Laboratory of High Active Traditional Chinese Drug Delivery System, Chongqing Medical and Pharmaceutical College, Chongqing 401331, China
[3] School of Pharmaceutical Sciences, Chongqing University, Chongqing 401331, China
* Correspondence: fuqifeng1990@163.com (Q. F.), znxia@cqu.edu.cn (Z. X.), raoli0728@163.com (L. R.)
† These authors contributed equally to this work.

Table of contents

1. FESEM images of the cross-sections of PDA-SP@capillary from the same batches
2. FESEM images of the inner wall of PDA-SP@capillary after washing treatment with HCl (pH 2.0) and NaOH (pH 10.0) solutions for 12 h
3. FESEM images of the inner wall of PDA-Tris@capillary from the same batches
4. Effect of the number of repeated coatings of PDA-SP@capillary and PDA-Tris@capillary on their EOF mobilities at pH 8.0
5. EOF stability of PDA-SP@capillary and PDA-Tris@capillary at pH 8.0 and pH 4.0
6. Effect of 1,3-PS concentration and sulfonation time on EOF mobilities of SPD@capillary
7. Electropherograms of six aromatic acids on bare capillary at different pH
8. Electropherograms of isoproterenol and ofloxacin on bare capillary at different pH
9. Electropherograms of isoproterenol on SPD@capillary at different buffer concentration, methanol concentration and applied voltage
10. Electropherograms of six aromatic acids of different runs on SPD@capillary

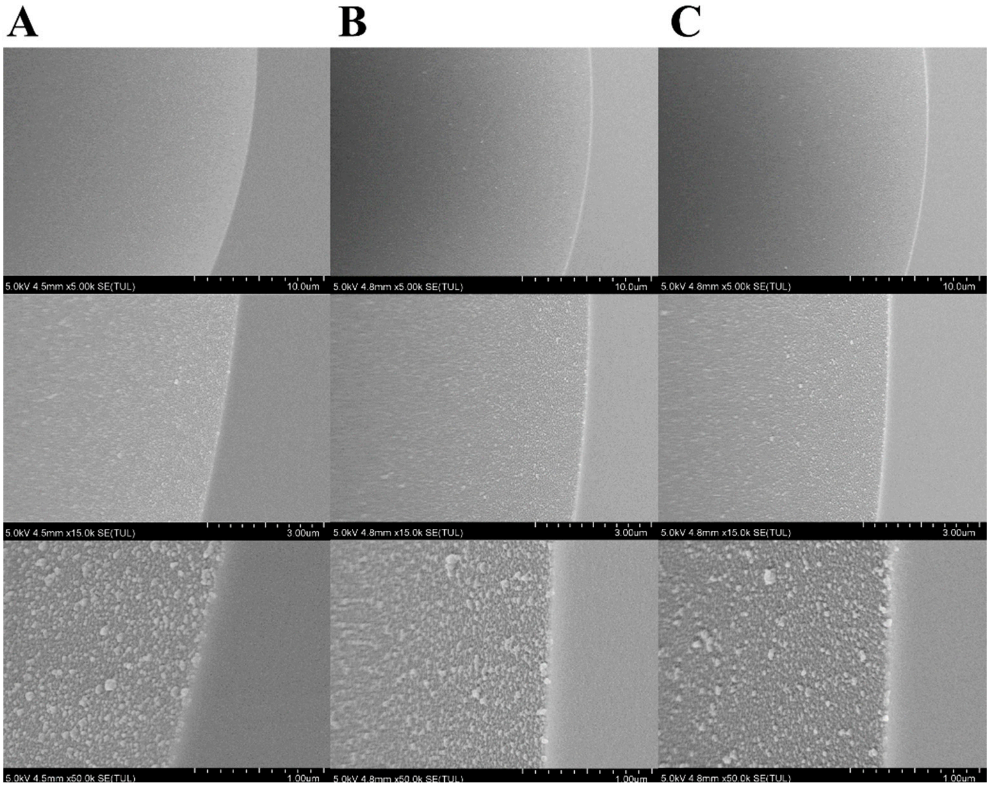

Figure S1. FESEM images of different cross-sections within a single PDA-SP@capillary.

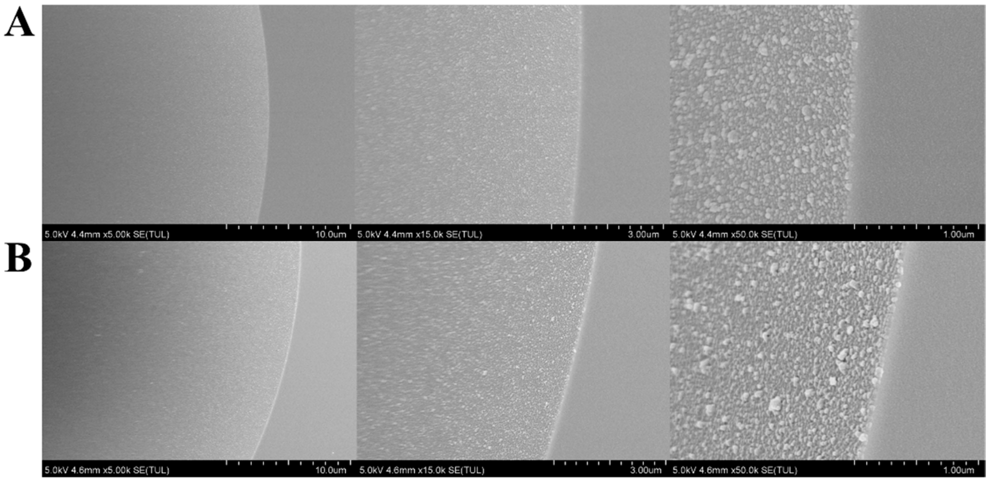

Figure S2. FESEM images of the inner wall of PDA-SP@capillary after washing treatment with HCl (pH 2.0) (A) and NaOH (pH 10.0) (B) solutions for 12 h.

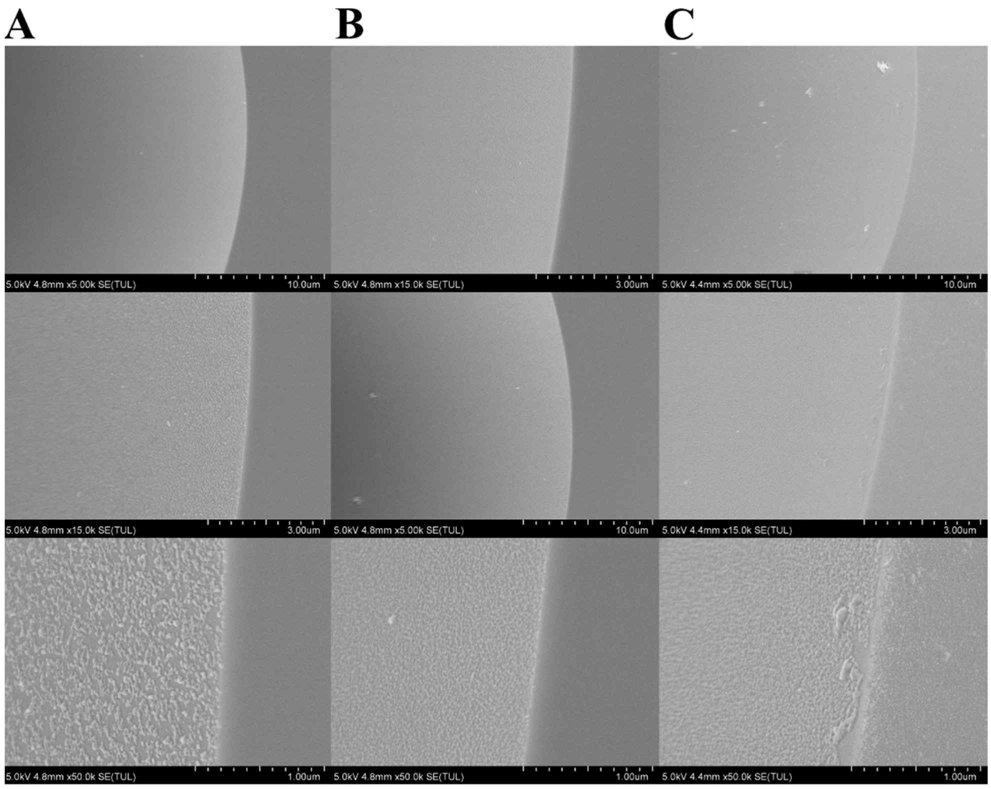

Figure S3. FESEM images of different cross-sections within a single PDA-Tris@capillary.

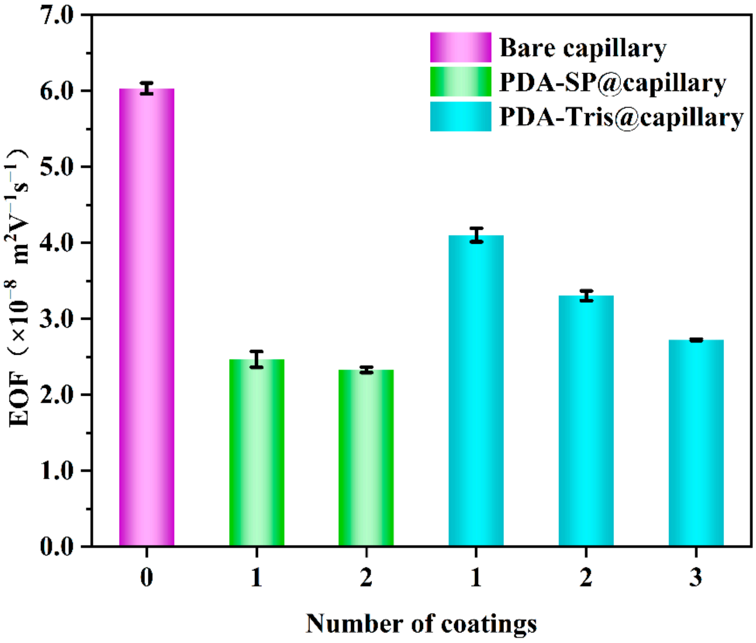

Figure S4. Effect of the number of repeated coatings of PDA-SP@capillary and PDA-Tris@capillary on their EOF mobilities at pH 8.0. All other CE experimental conditions are the same as Figure 4.

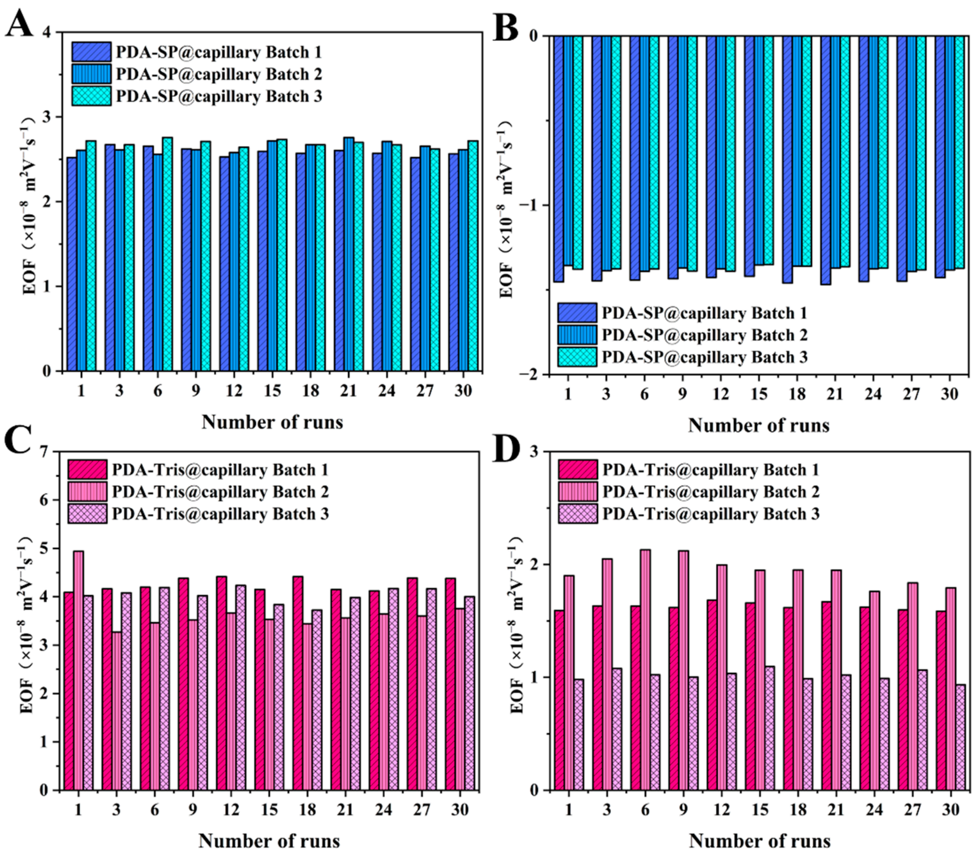

Figure S5. EOF stability of PDA-SP@capillary at pH 8.0 (A) and pH 4.0 (B) and PDA-Tris@capillary at pH 8.0 (C) and pH 4.0 (D). All other CE experimental conditions are the same as Figure 4.

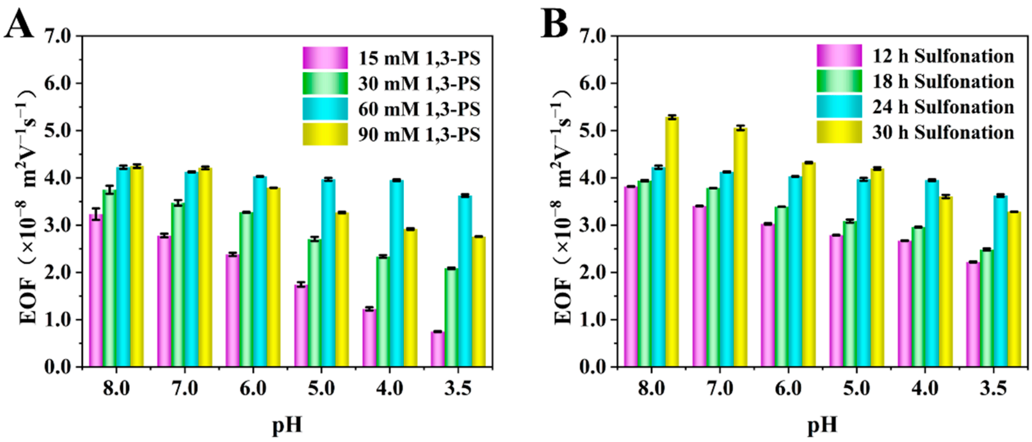

Figure S6. Effect of 1,3-PS concentration (A) and sulfonation time (B) on EOF mobilities of SPD@capillary. All other CE experimental conditions are the same as Figure 4.

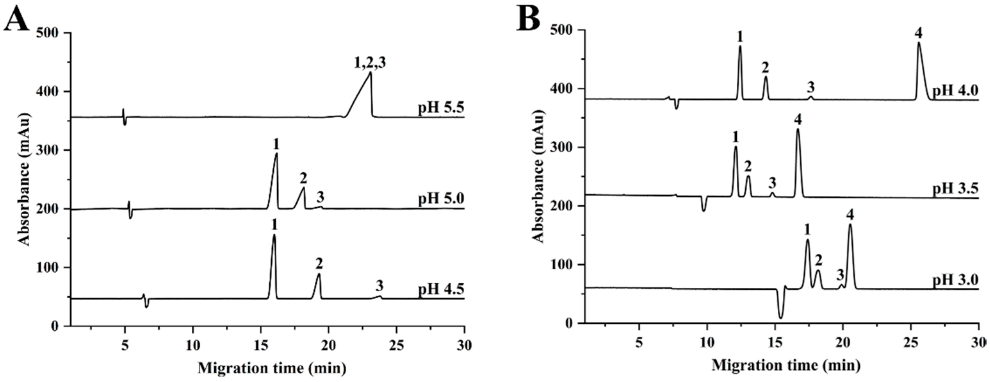

Figure S7. Electropherograms of six aromatic acids on bare column at different pH. All other CE experimental conditions are the same as Figure 5.

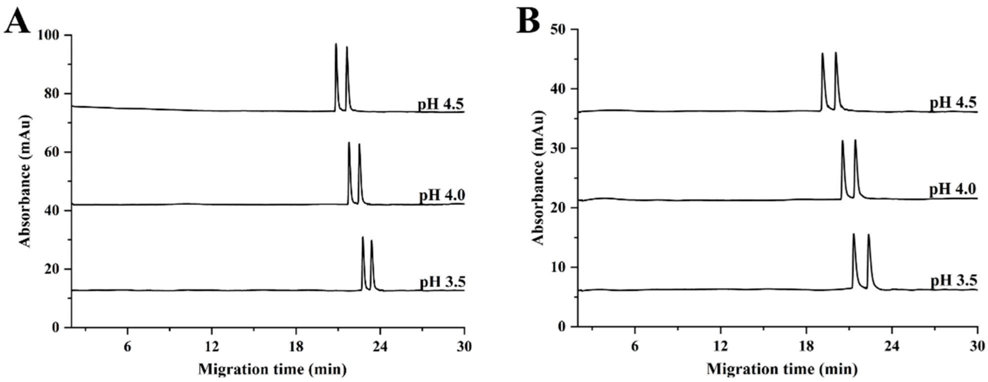

Figure S8. Electropherograms of (A) isoproterenol and (B) ofloxacin on bare capillary at different pH. All other CE experimental conditions are the same as Figure 6.

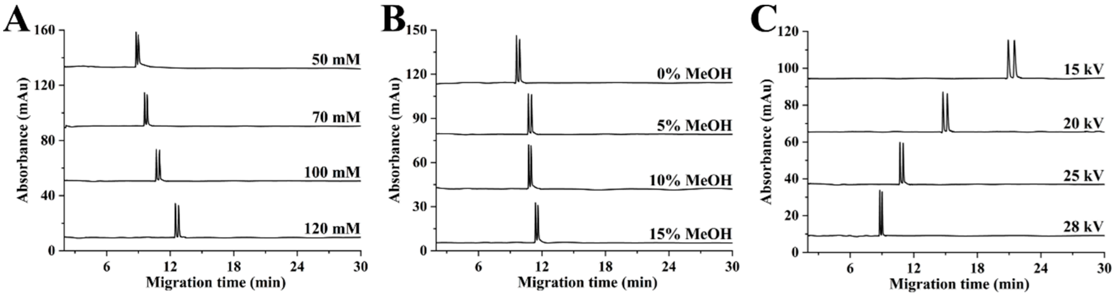

Figure S9. Electropherograms of isoproterenol on SPD@capillary at (A) different buffer concentration, (B) methanol concentration and (C) applied voltage. All other CE experimental conditions are the same as Figure 6.

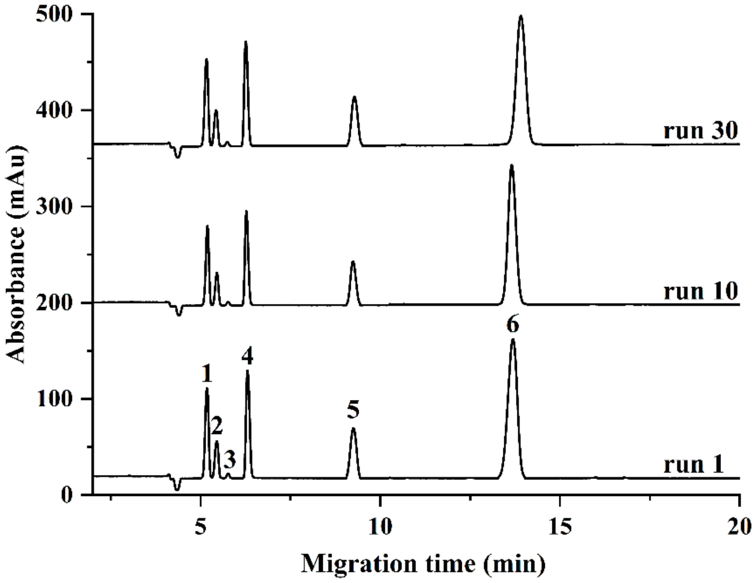

Figure S10. Electropherograms of six aromatic acids of different runs on SPD@capillary. All other CE experimental conditions are the same as Figure 5.
